# Molecular Approaches to Agri-Food Traceability and Authentication: An Updated Review

**DOI:** 10.3390/foods10071644

**Published:** 2021-07-16

**Authors:** Valentina Fanelli, Isabella Mascio, Monica Marilena Miazzi, Michele Antonio Savoia, Claudio De Giovanni, Cinzia Montemurro

**Affiliations:** 1 Department of Soil, Plant and Food Sciences, University of Bari Aldo Moro, Via Amendola 165/A, 70126 Bari, Italy; mascioisa@gmail.com (I.M.); monicamarilena.miazzi@uniba.it (M.M.M.); michele.savoia@uniba.it (M.A.S.); claudio.degiovanni@uniba.it (C.D.G.); cinzia.montemurro@uniba.it (C.M.); 2 Spin off Sinagri s.r.l., University of Bari Aldo Moro, Via Amendola 165/A, 70126 Bari, Italy; 3 Institute for Sustainable Plant Protection–Support Unit Bari, National Research Council of Italy (CNR), Via Amendola 122/D, 70126 Bari, Italy

**Keywords:** molecular traceability, authentication, agri-food, molecular markers, DNA barcoding, isothermal amplification, sequencing

## Abstract

In the last decades, the demand for molecular tools for authenticating and tracing agri-food products has significantly increased. Food safety and quality have gained an increased interest for consumers, producers, and retailers, therefore, the availability of analytical methods for the determination of food authenticity and the detection of major adulterations takes on a fundamental role. Among the different molecular approaches, some techniques such as the molecular markers-based methods are well established, while some innovative approaches such as isothermal amplification-based methods and DNA metabarcoding have only recently found application in the agri-food sector. In this review, we provide an overview of the most widely used molecular techniques for fresh and processed agri-food authentication and traceability, showing their recent advances and applications and discussing their main advantages and limitations. The application of these techniques to agri-food traceability and authentication can contribute a great deal to the reassurance of consumers in terms of transparency and food safety and may allow producers and retailers to adequately promote their products.

## 1. Introduction

The major worries of consumers concern the origin and the safety of the food they buy. The increased awareness of the value of food quality induces the consumer to ask for transparency from food companies. At the same time, companies must be able to certify the content and origin of their products with the aim of protecting the consumer against fraud and adulterations. In this scenario, traceability and authentication are fundamental tools for reassuring consumers in terms of transparency and food safety and allowing producers to gain awareness of the value of their products. Traceability lets the tracking of the source of a food at any point in the production chain enabling the quality-control processes and cutting down the production of unsafe or poor-quality foods [1]. Food authentication is the process through which a food is tested to verify if it complies with the description contained in its label [2].

Traceability and authentication are integral components of the food safety and defense system and represent fundamental components of the food supply chain. A reliable authentication and traceability system can constitute an essential instrument for the protection of consumers, reducing the chance of people consuming adulterated or contaminated foods, and increasing supplier control and process safety. Consumers showed limited knowledge about the importance of authentication and traceability of food products [3,4], making essential the dissemination of the potential and reliability of tracing methods with the purpose to increase people’s awareness of the role of food surveillance in health protection and the truthfulness of traceability information.

A wide variety of analytical methods for food traceability and authentication have been developed and tested [5]. Each method allows obtaining specific information on food composition and characteristics such as geographical origin, presence of adulterants, and species or varieties used in the production process. Among these analytical methods, the molecular approaches show some important advantages such as accuracy, sensitivity, and high reproducibility. Moreover, these methods are not affected by environmental changes, harvesting period, storage condition, and manufacturing process [6]. 

In the last decades, the demand for molecular tools for food authentication and traceability has significantly increased. This is mainly due to increasingly stringent legislation in the food sector and the market strategies aiming to assess a uniform and reliable control of the whole food chain from the field to the market and to ensure that consumer choices correspond to their expectations [7]. In this context, the European Union established two levels of recognition of food products: Protected Designation of Origin (PDO) and Protected Geographical Indication (PGI) with the purpose to protect the typical and local products and help consumers in choosing authentic food products and avoiding food frauds [8]. The DOP mark recognizes foods whose main characteristics depend on the territory of origin and the adherence to strict production rules. The IGP mark is attributed to a food that has a specific quality dependent on the specific geographical area of production. The availability of molecular analytical approaches is fundamental in the assessment of the conformity of PDO/PGI labels and the detection of not declared components.

Among the molecular analytical methods, some techniques such as the molecular markers-based approaches are well established, while some innovative approaches such as isothermal amplification-based methods and DNA metabarcoding have only recently found application in the surveillance of agri-products. Different authors have reviewed the most commonly used molecular methods for agri-food authentication [5,6,9,10,11], however, none of them have described the most recent and advanced techniques in detail and the potential of these methods in traceability and authentication processes. 

In this review, an overview on the principal analytical methods for agri-food authentication and traceability was provided, focusing in particular on the molecular approaches. We describe some of the proven and widely tested molecular approaches such as molecular markers-based methods, showing their latest applications in agri-food surveillance. Moreover, we explore the most recent technologies describing their potential and prospects in food authentication and traceability. Finally, the advantages and limits of each approach are described and discussed.

## 2. Analytical Methods for the Traceability and Authentication of Food Deriving from Plant Species

In the last twenty years, an exponential growth of studies on methods for the traceability of animal- and plant-based food has been observed [12,13]. For animal-based food, the main frauds concern the substitution of an ingredient and the animal’s geographical origin. In these cases, the analytical approaches are mainly based on vibrational spectroscopic techniques for the identification of the geographical origin and DNA typing of animal species [12]. For plant-based food, the fraudulent practices are highly disparate. The mismatch between product origin and geographical origin declared on the food label, the adulteration and contamination of product, the use of different species or different varieties compared with those declared on the label and the level of an additive higher than that permitted in a specific food are the most common frauds. Traceability approaches used for agri-foods are varied. Table 1 shows a list of the principal physico-chemical approaches used for plant-based food product traceability and authentication and the most recent reviews published for each method.

Chromatography allows for the separation and quantification of macro- and microcomponents in food products. The most widely used chromatographic techniques are high-performance liquid chromatography (HPLC) and gas chromatography. Both methods have been successfully used for the identification of the geographical origin of sweet cherry cultivars [27]. For agri-products, HPLC is an effective method to detect the presence of adulterants, quantify the level of additives, and identify the geographical origin of the product. The HPLC technique has been efficiently used for the authentication of extra virgin olive oil, the detection of adulteration in fruit juice, and the identification of the geographic origin of coffee, tea, and wine [18]. Gas chromatography is mostly applied in volatile substances analysis and detection of contaminants like pesticides. Gas chromatography analysis was performed to identify the geographical origin of different kinds of plant-based food products [13,19].

Immunoassays are analytical tools based on the use of antibodies or enzymes as recognition elements to detect the presence of specific antigens. Enzyme-linked immunosorbent assay (ELISA) is the most used immunological method for food traceability. This technique is mostly used for the detection of pesticide residues in food-borne matrices [28,29].

Spectroscopic techniques are fast and inexpensive methods based on the use of radiated energy to analyze the properties of a specific element. They have been widely used for different purposes including agri-food traceability. Fluorescence spectroscopy is a non-invasive and relatively inexpensive technique. However, it is less used compared to other spectroscopic methods due to its low detection limit. Despite this, fluorescence spectroscopy has been successfully used to detect adulteration in edible vegetable oils [30]. Vibrational spectroscopy is a widely used spectroscopic technique in the food sector. A wide array of vibrational spectroscopic methods including near-infrared (NIR), Fourier transform infrared (FTIR), and Raman spectroscopy have been used for the detection of adulteration and determining the authenticity of food products [14].

Nuclear magnetic resonance (NMR) allows for the identification of the composition of complex matrices of foodstuffs. The amount of any component in a mixture can be assessed with high precision. In the last years, NMR has been widely used for geographical traceability of agri-food products. This technique has been efficiently applied to the traceability of balsamic vinegar, saffron, coffee, and tomato [25], and recently to discriminate the origins of different species including rice, lentil, and citrus [31,32,33].

Among the most efficient methods for food authentication are the mass spectrometry (MS) techniques. A wide array of MS applications is available for food traceability and safety purposes such as the detection of contaminants, the composition, and the origin of a product [15]. Two MS techniques, isotope ratio mass spectrometry (IRMS), multi collector–inductively coupled plasma–mass spectrometry (MC-ICP-MS), are commonly used for the analysis of isotopic ratios in food matrices. The isotopic ratios are widely used in food authentication and traceability because they change with the area of origin of the product, climatic conditions, characteristics of soil, and agricultural practices. The most commonly used isotope ratios of elements for traceability of agri-products are ^13^C/^12^C and ^15^N/^14^N, influenced by climate condition and agricultural practices; ^2^H/^1^H and ^18^O/^16^O, affected by the area of origin; and ^34^S/^32^S, influenced by geology [34]. Several studies have applied the analysis of isotopic ratios to identify the origin of agri-products [16].

Usually, the food traceability and authentication methods based on physico-chemical analysis are used in combination with each other in order to reach maximum sensitivity and reliability. The combined use of gas chromatography with mass spectrometry allows for accurate qualitative and quantitative analyses of complex mixtures providing noteworthy results in the surveillance of agri-products [17]. A recent study showed that the combined analysis of stable isotopes, elemental composition, and chemical markers was demonstrated to be highly effective in the determination of the geographical origin of a product [35]. 

Although over the years these analytical methods have been proven to be highly efficient and reliable in the identification of the geographical origin and potential adulterants fraudulently added to a product, they show remarkable limitations in the detection of contaminant species and in unmasking the use of varieties not declared in the product label. Additionally, physico-chemical approaches have been shown to be highly reliable with fresh products while they tend to lose effectiveness in the analysis of processed foods. These limitations are overcome by the use of molecular methods to food traceability.

## 3. Molecular Approaches to Agri-Food Analysis

DNA is a stable molecule present in all living organisms and each organism’s DNA sequence is unique, enabling the distinguishing of the species and varieties used to produce a specific food. Moreover, DNA can also be recovered in enough quality and quantity in heavily processed food matrices. Thanks to the recent advancements in molecular biology and genetics, molecular approaches have become powerful and widely used methods for the authentication of agri-food products and for tracking the raw materials across the whole industry process. Along with the most widespread and experienced molecular marker-based methods, the more recent isothermal amplification-based methods, digital PCR techniques, and NGS-based approaches appear to be very promising in the traceability of a wide range of fresh and processed agri-foods. Table 2 shows a list of the most recent studies on agri-food authentication and traceability using DNA-based approaches.

### 3.1. Molecular Marker-Based Methods

Molecular marker-based methods are the most widely used techniques for food traceability. The main reasons are the reduced amount of template DNA required for marker detection, the chance to analyze simultaneously multiple target regions, and the possibility of obtaining both qualitative and quantitative information. In most cases, PCR-based methods are used to detect molecular marker variations [9]. PCR is diffusely employed in all molecular biology laboratories and does not require highly qualified personnel. Moreover, the low cost of the equipment and reagents makes PCR-based detection the easiest and most inexpensive method for molecular authentication and traceability of agri-products. The types of molecular markers most used for traceability purposes are microsatellite or Simple Sequence Repeat (SSR) and Single Nucleotide Polymorphism (SNP). They are highly informative due to their large number and even distribution throughout the genome and can highlight both inter and intra-species diversity [10].

#### 3.1.1. Simple Sequence Repeats (SSR)

**Figure 1:**
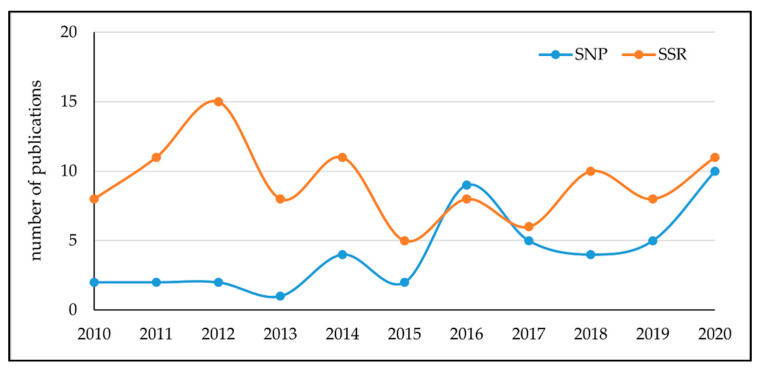
Number of publications per year in traceability and authentication of agri-foods through SSR and SNP markers. Data were obtained by searching the Scopus document archive (https://www.scopus.com; accessed on 8 June 2021) for English language articles for years between 2010 and 2020 using the following search terms: (SSR) AND (authentic*), (SSR) AND (traceability), (SNP) AND (authentic*) and (SNP) AND (traceability) and selecting only publications related to the agri-food sector and relevant to authentication and traceability processes.

Over the last ten years, the number of works based on the use of SSR for agri-food traceability and authentication has progressively reduced, together with an increase in papers employing the more abundant and stable SNP markers (Figure 1), nevertheless, SSR remains the most widely used marker for molecular traceability. Simple sequence repeats are tandem repeated motifs of 2–6 bp flanked by highly conserved sequences. The polymorphism is due to the different number of repeats in the microsatellite region, and can be easily detected by PCR. Their high reproducibility and polymorphism degree make them a marker of choice for many applications including varietal identification and adulteration detection [66].

Recently, SSRs have been efficiently used for the traceability of cocoa in beans and liquor [67], evaluations on trueness-to-type of raspberry [68] and olive [69] varieties, and to trace monovarietal and polyvarietal wines along the entire production chain [36]. Microsatellite markers have also been shown to be effective in tracing species characterized by a reduced diversity such as zucchini [70]. The most common approach involves the amplification of the regions of interest followed by fragment size evaluation through capillary electrophoresis. Nevertheless, the analysis of amplicons by the high resolution melting (HRM) assay was revealed to be highly effective in the authentication of PDO sweet cherry products [71] and the detection of adulteration in lentil [72]. Besides, the SSR-HRM technique allows for the authentication and traceability of processed food such as olive oil and wine. In particular, the combined use of SSR markers and HRM allows for distinguishing the varietal composition of olive oil and wine blends determining a limit of detection for adulteration included between 1% and 2.5% [37,38,39,73]. Moreover, microsatellite detection through real-time PCR enables the quantification of a specific contaminant. Pasqualone et al. [74] identified the common wheat contamination in durum wheat semolina and bread through the detection of genome D-specific SSR. The authors observed a detection limit of 3% and 5% for semolina and bread, respectively, by qualitative PCR lowered to 2.5% by real-time PCR.

#### 3.1.2. Single Nucleotide Polymorphism (SNP)

Single nucleotide polymorphisms (SNPs) are variations in the DNA sequence involving a single base. They are the most abundant and ubiquitous markers in any living organism and their diallelic nature offers a lower error rate in allele calling compared with other molecular markers. Moreover, SNPs identification does not require DNA separation by size, and it is suitable for automation, making the analysis quick and reproducible.

SNPs are widely used in the traceability of animal-based foods, especially in the genetic authentication of meat [75], while only a few works are available in the agri-food sector, however, their use in this field has increased significantly in the last years and it is expected to keep growing in the future (Figure 1). The development of SNP-based approaches to agri-food traceability is encouraged by the increasing number of SNP catalogs mostly derived by GBS analysis. These panels are available in different species of agri-food interest such as grapevine, olive, pulses, cacao, and coffee [76,77,78,79,80]. Most of the works using a SNP-based traceability approach have focused on olive oil analysis [40,41], however, SNPs have been efficiently employed in differentiating Arabica and Robusta coffee varieties [81], in the authentication of Portuguese wine [42], and in the identification of Nebbiolo variety in musts and wines [43]. 

SNP identification is suitable for different detection methods such as single-base primer extension, cleaved amplified polymorphic sequences assays (CAPS), HRM, and sequencing techniques. Recently, an innovative system for wine authenticity based on the use of a biosensor as the system of SNP detection was developed by Barrias et al. [44]. DNA-based biosensors use DNA strands as probes for sensing DNA targets, distinguishing among samples differing for a single nucleotide in their sequence. The authors demonstrated the ability of the system to discriminate the varieties present in leaf, must, and wine samples, showing the promising application of this technique in SNP-based agri-food authenticity. Additionally noteworthy is the SNP genotyping system based on the use of a nanofluidic array. This system consists of the use of integrated fluidic circuits for high-throughput real-time PCR, allowing for the reliable analysis of multiple samples in a short time using small quantities of DNA. The nanofluidic SNP protocol has been successfully applied for cultivar authentication and identification of the adulterant varieties in cacao beans [82], discrimination of 40 tea varieties [83], and cultivar differentiation of coffee beans [45]. 

The rapid advances of next generation sequencing technologies have allowed for the automation of SNP detection, making the analysis based on this marker more rapid and reliable [84]. The employment of innovative sequencing approaches will allow the further spread of SNP-based approaches in the safeguarding of agri-food safety and quality.

### 3.2. Single Region Approaches

For some applications, the investigation focuses on a specific and well-known target DNA region. The analysis can be performed with the purpose to amplify a DNA sequence of a specific species or variety, taking advantage of peculiar differences in that region (e.g., indels). Conversely, PCR primers can be designed in a specific conserved region to amplify a sequence characterized by a certain polymorphism among species. This is the case of the DNA barcoding approach, representing an important tool for food traceability and authentication [85]. Isothermal amplification-based methods seem to be very promising and represent a novel group of nucleic acid amplification technologies that are simple and highly specific. Recently, these strategies have been successfully applied in the agri-food authentication sector.

#### 3.2.1. Species-Specific Primer PCR

The presence of differences in nucleotide sequence or indels allows for the design of primers specific for a species or a variety. The detection of an amplification product makes possible the identification of adulterant species or variety in a particular food-borne sample. This approach has been widely used for the detection of common wheat in durum wheat-based products such as pasta or durum wheat bread. The identification of the presence of common wheat can be addressed by the detection of a sequence-specific of the D-genome, which is present in hexaploidy wheat but absent in durum wheat. Sonnante et al. [86] focused on the microsatellite region GDM111 to develop a quantitative method to detect the common wheat contamination in semolina, bread, and pasta products. The method was revealed to be effective up to a limit of 1% common wheat contamination. Matsuoka et al. [87] employed the *Starch Synthase II* (*SS II*) gene, coded on wheat A, B, and D genomes. The authors took advantage of some differences in the *SS II-D* gene to set up a quali-quantitative method for the detection of common wheat in blended flour. Silletti et al. [46] used a tubulin-based polymorphism to develop an assay specific for the detection of common wheat adulteration in pasta and flour. Through a DNA-based multiplex detection tool, Voorhuijzen et al. [88] were able to simultaneously test 15 different grain ingredients within one food with high accuracy. 

In recent years, the development of techniques based on digital PCR (dPCR) has made the detection of a contaminant in food much faster and easier [89]. Digital polymerase chain reaction enables absolute quantification of a target nucleic acid in a sample even when the target is present at a very low number of copies. dPCR works by partitioning DNA fragments into thousands of independent droplets or chips, making it possible to directly count the number of target molecules through Poisson statistics [90]. dPCR has been widely used in the field of genetically modified organism (GMO) monitoring [91] and for pathogen diagnostics [92]. Moreover, this technique was also revealed to be very reliable and accurate in food safety and adulteration control. Pierboni et al. [93] efficiently applied droplet digital PCR to detect the presence of peanut and soybean allergens in mill and bakery products and demonstrated the usefulness of this technique for the food safety of allergic populations. More recently, Morcia et al. [47] developed a duplex chip digital PCR assay able to identify and quantify common wheat presence along the whole pasta production chain. The authors found that the limit of detection of the proposed method was 0.3% common wheat contamination, whereas the limit of quantification was found at the 1.5% level. Duplex droplet digital PCR and chip digital PCR were also revealed to be effective in the quantitative detection of kidney beans in lotus seed paste [48]. Generally, lotus seed paste is adulterated with cheaper ingredients such as common beans, making the detection method based on digital PCR extremely useful in revealing fraudulent substitutions or adventitious contaminations.

#### 3.2.2. DNA Barcoding

DNA barcoding was developed by Hebert et al. [94] and is based on the analysis of variability within a specific genomic region called the “DNA barcode”. This method represents an effective approach to food traceability and authenticity since it does not require extensive knowledge of the genome sequence of each organism and allows for the identification of more than one species at the same time. In animal-based food traceability, the barcoding is frequently based on the amplification of the *cytochrome oxidase* gene. In terrestrial plants, plastidial genes *rbcL* and *matK*, the trnH-psbA intergenic spacer and nuclear ITS2 sequence are mostly used as barcode regions [85]. DNA barcoding efficiency has been widely demonstrated in discriminating spices species such as nutmeg [49]. Recently, the analysis of trnH-psbA spacer and ITS2 sequence revealed them to be effective in the authentication of ginseng products [95] and the identification of adulterants in coffee and almond [50,96].

Frequently, DNA barcoding is employed coupled with high resolution melting (HRM) analysis (Bar-HRM). It consists in the amplification of a short DNA barcoding sequence and target region detection through HRM based on the distinctive melting behavior due to differences in DNA sequence. In the last years, the Bar-HRM strategy has found a large spread in agri-food surveillance. Bosmali et al. [97] set up a fast and cost-effective Bar-HRM method for PDO saffron authentication. The proposed approach was revealed to be highly effective in terms of specificity and sensitivity compared to other methods. A similar approach was used for the authentication of commercial sea buckthorn products [98]. More recently, Bar-HRM was employed for the authentication of several commercial tea products and detection of the presence of cashew DNA in the tea products [51], identification of common nut adulterants in walnut milk beverage [52], and the quantitative detection of Robusta traces in Arabica coffee products [53]. The great potential of the Bar-HRM technique has been widely demonstrated by Ballin et al. [99]. In this study, a DNA profiling platform for species authentication throughout the plant kingdom was developed through a multiplexed Bar-HRM approach. Distinct melting profiles were obtained for species originating from 29 different families spanning the angiosperms, gymnosperm, mosses, and liverwort, demonstrating the ability of the proposed approach in discriminating a large number of species without a priori knowledge of the species’ DNA sequence.

DNA barcoding-based approaches in agri-food authentication and traceability are promising thanks to the great advances made in molecular biology techniques that allow us to combine the detection of a specific barcode sequence with modern technologies such as nanotechnologies. Based on this principle, Valentini et al. [54] developed an easy and inexpensive approach called “Nanotracer”, which is able to detect the presence of a specific species-DNA in a food sample through a colorimetric response. The proposed approach is based on an asymmetric PCR amplification of a short barcode region, yielding a single-strand amplicon that is readily hybridizable to induce a color change due to the presence of DNA-functionalized gold nanoparticles. This method offers a rapid and naked-eye authentication test, and its implementation in the agri-food sector will provide an efficient system for food surveillance in the future.

The potential of the DNA barcoding strategy can be exploited through the sequencing of amplicons. The obtained sequence can be used to differentiate and univocally identify the species present in a food sample through a comparison with specific molecular databases. Recently, Sanger sequencing of specific DNA barcode regions was efficiently used for authentication of small berries in fruit products [55] and the construction of a DNA barcode library for the traceability of Chinese herbs [100]. However, the high costs and the limited number of samples that could be analyzed at the same time, along with the necessity of high-quality DNA, led Sanger sequencing to be supplanted by the next generation sequencing (NGS) technologies, which offer a much higher throughput through a less expensive and less time-consuming procedure.

The adoption of a universal barcode shows evident limits at the cultivar level, where genetic variability is limited. To overcome these limits, the ultra-barcoding methodology was proposed [101] to obtain a varietal identification. This strategy is based on the sequencing of the whole plastidial genome and a portion of the nuclear genome through NGS technologies. Ultra-barcoding has been shown to be a highly reliable strategy in cacao authentication [102]. 

The use of the DNA barcoding method in the agri-food sector is supported by the availability of the Barcode of Life Database (BOLD) coordinated by the International Barcode of Life Project [103]. This database contains a reference library for all living species, allowing the identification of more than 300,000 species on the base of the barcode sequence. Moreover, it includes a comprehensive registry of primers useful in the generation of barcode sequences. BOLD is a reliable resource for the exploitation of the potentiality of the DNA barcoding approach in food authenticity and safety. 

#### 3.2.3. Isothermal Amplification-Based Methods

Isothermal amplification-based techniques represent a promising alternative to classical PCR since they achieve rapid and efficient detection of a nucleic acid target without requiring the use of a thermocycler. These methods allow the amplification of a specific region in an exponential manner at a constant temperature. Over the last decade, various techniques based on isothermal amplification have been developed; although their features can vary among the different methods, they share some characteristics such as the use of a polymerase with strand-displacement activity. Some of the isothermal amplification techniques mostly used in agri-food surveillance are rolling circle amplification (RCA), multiple displacement amplification (MDA), recombinase polymerase amplification (RPA), and loop-mediated isothermal amplification (LAMP). These methods are mostly used in the detection of various micro-organisms, representing an important instrument to control food-borne diseases and safeguard food safety and quality [104]. Furthermore, they were also revealed to be highly sensitive and efficient in agri-food authentication and traceability. RPA in combination with ELISA has been shown to be highly effective in the detection of allergens such as hazelnut, peanut, and soybean as well as undeclared food ingredients [105]. Recently, Zhao et al. [56] proposed a novel analysis based on the combined use of RPA and lateral flow device (RPA-LFD) for saffron authentication. This rapid assay was revealed to be highly sensitive and specific, with no cross-reaction with common saffron adulterants. 

Among the isothermal amplification-based methods, LAMP is the most widely used. This technique employs four to six different primers able to recognize six to eight different sequences of a target region, allowing the synthesis of large amounts of DNA in a short time. The amplification products are stem-loop DNAs with different inverted target repeats; these products can be detected with different methods including real-time assay and naked-eye detection through DNA-binding dyes or colorimetric indicators [106]. The high specificity, efficiency, and simplicity of the LAMP method has led to its application in the identification of different micro-organisms including food-related pathogens [107]. This approach is also suitable for the detection of GMOs through the employment of commonly used promoters or marker genes as LAMP targets [108]. Recently, LAMP has also assumed a relevant role in agri-food surveillance for the identification of specific species or even a variety in a specific food product. This approach has been used to authenticate saffron and discover its adulterants such as safflower and turmeric [57]. Cibecchini et al. [58] set up a portable colorimetric LAMP-based method to detect the presence of a specific wheat variety (Aureo) in grains and flours. Hu and Lu [59] developed a device for the specific detection of pomegranate, apple, and grape DNA present in fresh fruit juice. The authors combined DNA extraction and LAMP reaction in a hybrid paper/polymer-based lab-on-a-chip platform, allowing for the quick detection of a specific species in a juice sample through the use of a fluorescent dye. In the future, this method is expected to play an important role in the field of agri-food authentication and traceability.

### 3.3. Next Generation Sequencing-Based Methods

DNA sequencing represents the easiest way to detect multiple species and varieties present in a specific food-borne sample. Traditional Sanger sequencing allows for the detection of a specific DNA region at a time. Although cloning may improve resolution, it requires numerous steps and is very time-consuming. Moreover, Sanger sequencing is a relatively slow method, producing reads with a length not exceeding 900 bp [109]. Next generation sequencing (NGS) is a high throughput technique enabling the generation of different quantities and lengths of DNA sequencing. The different approaches are commonly grouped based on the length of reads produced during the sequencing. Therefore, we distinguished between short-read and long-read sequencing methods defined as second- and third-generation technologies, respectively. 

The short-read sequencing approaches such as sequencing by synthesis and ion semiconductor sequencing were the first NGS techniques to be developed. Illumina is the current leader for the short-read sequencing approach. This technique is based on the peculiar bridge amplification method and the sequencing by synthesis strategy, which generates long-reads up to 300 bp [110]. Another popular short-read strategy is the ion semiconductor sequencer Ion Torrent based on the use of a dedicated sensor that acts as a highly sensitive pH meter, which detects the hydrogen ion release associated with nucleotide incorporation into the growing strand. For authentication of processed foods, the short-read-based sequencing strategies are preferable since DNA recovered from these matrices is usually highly degraded.

Third-generation strategies are quite recent techniques that enable overcoming many of the limitations of short-read sequencing through the sequencing of a single DNA/RNA molecule and generating reads with a length between 1 kb and 2 Mb [110]. The main long-read approaches are the single-molecule real-time sequencing (SMRT) and the nanopore sequencing. Despite the great potential of these techniques, their use is extremely limited in the food traceability sector.

Although the use of NGS technologies has spread in several diagnostics and research sectors, their use in the field of agri-food molecular traceability remains limited. A possible explanation is that NGS technologies present high costs and require extensive computational power. In addition, these strategies require high-quality DNA, which is not always possible to recover from highly processed foods. Nevertheless, a certain number of studies on agri-food traceability and authentication through NGS-based approaches have been published. There are basically two adopted strategies: whole metagenome sequencing and DNA metabarcoding.

#### 3.3.1. Whole Metagenome Sequencing

Whole metagenome sequencing (WMS) allows scanning for several species simultaneously even when these are present in a small quantity in a food matrix [111]. This approach is widely used in the food security sector to identify and characterize complex microbial communities in food samples [112]. An important advantage of using WMS in food-borne hurtful microbial detection is the possibility of also detecting non-culturable pathogens; moreover, the production of draft genome sequences of the bacteria responsible for food-borne alerts is also possible, allowing for the identification of contamination sources [113]. Likewise, WMS can be employed to trace specific species and even varieties with very high sensitivity and specificity. The analysis of whole genomes allows for the authentication and detection of non-approved species. Complex food matrices can be analyzed, and the detected reads assigned to corresponding organisms by comparison with “ad hoc” databases. 

A software pipeline, called AFS (All-Food-Seq), was developed to quantitatively measure the species composition in food-borne samples. This pipeline takes advantage of the deep sequencing of total DNA, allowing for the identification of species components through the mapping of reads to publicly available reference genome sequences and the quantification of species proportions based on a sequence read counting approach. This method has been successfully applied for the traceability and authentication of different animal- and plant-based foods [111].

More recently, Haiminen et al. [114] set up a bioinformatic pipeline, FASER (Food Authentication from SEquencing Reads), to resolve the relative composition of mixtures of eukaryotic species using RNA or DNA sequencing. Moreover, they developed a comprehensive database including more than 6000 plants and animals that may be present in food. FASER was revealed to be a highly sensitive and accurate method to detect fraudulent substitutions or contaminations in the most disparate food matrices.

Whole metagenome sequencing has been proved to be very effective in the identification and authentication of herbal products [60] and the detection of contaminants in food processed samples [61]. In the latter work, the authors combined metagenomic sequencing and an alignment-free k-mer based approach for the identification of plant DNA in processed samples. In particular, they demonstrated that lupin DNA can be individuated in controlled mixtures of sequences from the target and closely related non-target species, showing that lupin-specific components are detectable in baked cookies containing a minimum of 0.05% of lupin flour in wheat flour.

The whole chloroplast genome can be sequenced as an alternative to nuclear DNA for food authentication purposes. This is particularly useful in highly processed agri-foods since organellar DNA is present in high copy numbers compared to nuclear DNA, preventing degradation occurring during the production process. The sequencing of chloroplast genome produces reads that can be compared to specific databases containing complete chloroplast genome sequences such as the GenomeTrakrCP, which is publicly available at the National Center for Biotechnology Information (https://www.ncbi.nlm.nih.gov/bioproject/PRJNA325670/; accessed on 24 May 2021) [115]. This approach has been demonstrated to be highly effective by several authors [62,63].

#### 3.3.2. DNA Metabarcoding

The DNA metabarcoding approach combines the high throughput sequencing strategies with DNA barcoding, allowing the analysis of multiple amplicons corresponding to different barcode regions by sequencing them in parallel. The general strategy is based on extracting the whole DNA from certain foods, amplifying a specific barcode region whose dimensions can vary from 120 up to 600 bp, sequencing the corresponding amplicon, and analyzing the sequence using specific pipelines. This strategy is particularly suitable for highly processed foods since the DNA extracted from these matrices is usually degraded, making possible only the amplification of short regions [7]. Moreover, the DNA metabarcoding approach has also been demonstrated to be useful for quantitative analysis. In fact, differences in sequence reads abundance between species can be used to infer the corresponding differences in species abundance in a food sample [116]. 

The most commonly used plant barcode regions for DNA metabarcoding analysis are the nuclear ITS regions or the plastidial rbcL and psbA-trnH. In particular, the ITS1 and ITS2 regions have been used to identify plant components in herbal teas through their sequencing through two different platforms, Illumina and Ion Torrent, showing that both sequencing strategies are effective in qualitative and quantitative detection of different species [117]. Frigerio et al. [118] analyzed the sequence variability at DNA barcoding psbA-trnH and ITS and minibarcoding rbcL 1-B regions to trace medicinal and aromatic plants. Recently, a comprehensive ITS reference dataset called PLANiTS including all the ITS sequences of the Viridiplantae clade was developed [119]. The PLANiTS dataset represents a reliable first step toward an accurate standardization of plant DNA metabarcoding studies. 

The effectiveness of DNA metabarcoding in the agri-food authentication and traceability sector has been widely demonstrated in the authentication of polyfloral and monofloral honey [64,65,120]. In these cases, the metabarcoding approach allowed not only for the identification of the botanical composition of honey, but also to investigate its geographical origin based on the genetic characterization of pollen content.

Recently, Gostel et al. [121] developed microfluidic enrichment barcoding (MEBarcoding) for high-throughput plant barcoding, a cost-effective method based on the combined use of the Fluidigm Access Array and Illumina MiSeq. This study enabled them to build a highly comprehensive barcode database and demonstrated that the proposed approach is efficient in discriminating a very large number of species present in a food-borne matrix at the same time.

## 4. Advantages and Limits of Molecular Methods in Agri-Food Authentication and Traceability

A wide variety of analytical techniques for authentication and traceability of agri-food products have been developed and tested. For a long time, chemical and biochemical approaches have been used for the detection of specific components in foodstuffs; nevertheless, in the last few decades, molecular techniques have taken the upper hand in the food surveillance sector. DNA-based methods are mostly used for the identification and quantification of species and varieties composed of fresh or processed food. Indeed, DNA is present in nearly all the cells of a given organism and its sequence remains unchanged during all production phases. Instead, proteins and secondary metabolites may be influenced by growing conditions, harvesting period, and storage environment [6]. Moreover, DNA is a much more resistant molecule to industrial transformation compared to other biological components. On the other hand, physical fragmentation and chemical treatment can affect the yield, integrity, and quality of DNA [11]. For this reason, several protocols for DNA extraction from processed agri-food matrices were developed with the aim to recover a sufficient amount of good-quality DNA for subsequent analysis (Table 3). These protocols were optimized to extract DNA from a specific food-borne product with the purpose of maximizing the yield while minimizing the coextraction of enzymatic reaction inhibitors.

A valid alternative to nuclear DNA-based analysis is the use of approaches involving the chloroplast genome, which is present in high copy numbers in vegetal cells. Indeed, heavily industrial treatments can severely affect nuclear DNA quality and quantity, while this occurs to a lesser extent with chloroplast DNA due to its abundance [62,63]. 

Despite the significant advances that have been made in molecular techniques, innovative approaches are only partially used in agri-food authentication, while traditional molecular marker-based methods, whose effectiveness have been amply demonstrated, remain the approaches of choice. Regarding molecular marker-based methods, SNPs and SSRs are largely used nowadays because of their standardized and straightforward detection systems. These approaches are used mainly in the identification of plant varieties aiming to prevent fraudulent commercial activities. SNP and SSR application for food traceability and authentication offer several advantages: they have a high level of polymorphism, high reproducibility, and can be detected on a very small portion of DNA, which in the case of fragmented DNA may constitute an important advantage [127]. Moreover, recent technical advances in SNP detection have made this marker an election tool in food traceability. Indeed, modern sequencing technologies allow millions of SNPs to be processed, simultaneously making possible the analysis of several samples in extremely short times [128]. Nevertheless, being highly species-specific, the molecular marker-based methods require the knowledge of plant species putatively present in a food and access to the correct DNA sequence of interest. Therefore, their application is often limited to a single species [129].

Frequently, a food can contain several vegetal species and the availability of an instrument able to detect all the species simultaneously becomes necessary for traceability and authentication purposes. Approaches based on DNA barcoding represent an effective alternative to DNA fingerprinting methods in plant identification since they do not require the knowledge of the whole genome of an organism, being based on the exploitation of one or few genomic regions [11]. DNA barcoding shows two important advantages: the requirement to amplify a very short DNA region (a few hundred base pairs) and the widespread use of plastidial genome, which is more preserved during industrial processing [85]. Moreover, the availability of several plant DNA barcoding databases considerably simplifies species detection and identification [103,119,121]. Nevertheless, DNA barcoding presents some important limitations. First, only the species for which a reference is available can be identified; therefore, database incompleteness greatly affects the reliability of analysis [109]. Another important limit of DNA barcoding is that it can only be applied to identify monophyletic species, since polyphyletic and paraphyletic species do not display a clear barcode gap (i.e., a gap between frequency distributions between intra- and interspecific distances). The absence of a barcoding gap makes the definition of a threshold value to identify species impossible, generating either false negatives (species missed) or false positives (false species) [130]. This consideration makes evident the limitations of adopting a barcode-based strategy for cultivar distinction. Therefore, in some cases, a combined approach of molecular markers and DNA barcode would be the best strategy for an accurate and exhaustive authentication analysis [72,97].

Whole metagenome sequencing is the best strategy for authenticity, since it allows for the detection of additives, poisonous plants, allergens, and any other kind of adulterants fraudulently or accidentally added to a food-borne product. The main limitation of NGS-based methods in agri-food authentication is the obtainment of sufficiently high-quality DNA. This step is crucial to ensure that all DNA sequences present in a food-borne sample are properly identified [113]. A large number of DNA extraction protocols are now available for different kinds of foods including highly processed products (Table 3). These protocols take into account the specific features of a product implementing a series of steps aiming at the collection of a minimum amount of sufficient quality DNA on one hand, and the removal of inhibitors on the other. In some cases, the tuned protocol resulted in being highly effective in isolating DNA suitable for high throughput approaches [65,120]. Despite the great potential, the current use of NGS within the agri-food authentication and traceability sector is limited compared to the more established techniques. In the near future, the technological advances of NGS techniques, along with a cost reduction and more user-friendly options for analysis, will make these approaches increasingly widespread in food authenticity.

## 5. Conclusions

Agri-food traceability and authentication require reliable and accurate methods for the identification of plant species and varieties in a wide collection of fresh and processed food, without ambiguity. The possibility of being aware of the composition of a food has assumed increasing importance among consumers, thanks to the action of mass communication concerning the relevance “of knowing what one is eating”. Among the different traceability techniques, molecular approaches are gaining increasing interest due to their significant advantages compared to the physico-chemical approaches. 

There are many various molecular methods suitable for agri-food surveillance. Some of them such as the molecular marker-based approaches have been extensively experienced and used in the agri-food sector; several authors have described their main applications in detail. Here, we presented the advances of these approaches and their most recent employment in agri-food traceability and authentication. Moreover, we provided an extensive description of the most innovative approaches such as isothermal amplification-based methods and DNA metabarcoding, which have only recently found application in agri-food surveillance. We highlighted their potential and prospects by showing the latest works on traceability and authentication based on the use of these methods. Finally, the description of the main advantages and limits of each molecular method will represent an effective prompt for anyone who wants to find the best method to authenticate or trace a specific agri-food.

The wide panel of molecular techniques to traceability and authentication in the agri-food sector constitutes a powerful tool to protect both producers and consumers, ensuring consumer freedom of choice and improving the transparency of food production systems, therefore allowing honest producers to adequately promote their food products.

## Figures and Tables

**Table 1:** Summary of the most recent reviews about the principal methods based on physico-chemical analysis for agri-food traceability and authentication and the food matrices on which they are commonly used.

Analytical Method	Food Products	References
Vibrational spectroscopic techniques	Different agri-food products	Lohumi et al. [14]
Mass spectrometry techniques	Different agri-food products	Castro-Puyana and Herrero [15]
Stable isotope analysis	Cereals, wine, and vegetable oils	Zhao et al. [16]
Gas chromatography coupled with mass spectrometry	Wine, hazelnuts, barley, terebinth, olive oil, coffee, vegetables, and fruits	Dymerski [17]
HPLC	Olive oil, coffee, tea, wine, juice, fruit, nuts	Esteki et al. [18]
Gas chromatography	Wine, chocolate, coffee, saffron, vegetable oil, fruit	Nolvachai et al. [19]
Spectroscopic and spectrometric techniques	Wine, vegetable oils, coffee, wheat, nuts, rice, vegetables and fruits	Medina et al. [20]
ELISA	Different agri-food products	Wu et al. [21]
Fluorescence spectroscopy	Vegetable oils, cereals, vegetables and fruits	Ahmad et al. [22]
Spectroscopic techniques	Vegetable oils, coffee, wine, fruit juice	Esteki et al. [23]
Raman spectroscopy	Olive oil, coffee, wine, rice	Xu et al. [24]
NMR	Balsamic vinegar, saffron, coffee, tomato	Consonni and Cagliani [25]
Mass spectrometry techniques	Wine, fruit juice, olive oil, beer, coffee	Rubert et al. [26]
Spectroscopic and spectrometric techniques	Different agri-food products	Wadood et al. [13]

**Table 2:** List of the most recent studies on DNA-based methods applied in the traceability and authentication of agri-foods.

Technique	Agri-Food Product	Detected Species	References
SSR/capillary electrophoresis	Grapes, must, and wine	Grapevine (*Vitis vinifera* L.)	[36]
SSR/HRM and SNP/HRM	Olive oil	Olive (*Olea europea* L.)	[37]
SSR/HRM	Olive oil	Olive (*Olea europea* L.)	[38]
SSR/capillary electrophoresis and SSR/HRM	Must and wine	Grapevine (*Vitis vinifera* L.)	[39]
SNP/PCR-RFLP	Olive oil	Olive (*Olea europea* L.)	[40]
SSR/capillary electrophoresis and SNP/Sanger sequencing	Extra virgin olive oil	Olive (*Olea europea* L.)	[41]
SNP/HRM	Must and wine	Grapevine (*Vitis vinifera* L.)	[42]
TaqMan SNP Genotyping Assay	Must and wine	Grapevine (*Vitis vinifera* L.)	[43]
SNP/biosensor	Must and wine	Grapevine (*Vitis vinifera* L.)	[44]
SNP/nanofluidic array	Coffee beans	Coffee (*Coffea arabica* L. and *Coffea canephora* Pierre ex. A. Froehner).	[45]
Species-specific primer PCR/sequencing	Flour, pasta, bread, and cookies	Common wheat (*Triticum aestivum* L.)	[46]
Species-specific primer/digital PCR	Flour and pasta	Common wheat (*Triticum aestivum* L.)	[47]
Species-specific primer/digital PCR	Lotus seed paste	White kidney bean (*Phaseolus vulgaris* L.).	[48]
DNA barcoding/sequencing	Nutmeg mace	Nutmeg tree (*Myristica fragrans* Houtt)	[49]
DNA barcoding/capillary electrophoresis	Almond oil and almond kernels	Almond (*Prunus dulcis* Mill.)	[50]
Bar-HRM	Tea products	Tea (*Camellia sinensis* L.)	[51]
Bar-HRM	Nut species and walnut milk beverage	Walnut (*Juglans regia* L.), pecan (*Carya illinoensis* K. Koch), hickory (*Carya cathayensis* Sarg.), and peanut (*Arachis hypogaea* L.)	[52]
Bar-HRM	Raw seeds and ground coffee	Coffee (*Coffea arabica* L. and *Coffea canephora* Pierre ex. A. Froehner).	[53]
DNA barcoding/NanoTracer strategy	Saffron powder	Saffron (*Crocus sativus* L.)	[54]
DNA barcoding/sequencing	Berry fruit and fruit juice	Different taxa	[55]
RPA-LFD	Saffron powder	Saffron (*Crocus sativus* L.)	[56]
DNA barcoding/LAMP	Saffron powder	Saffron (*Crocus sativus* L.)	[57]
LAMP	Durum wheat products	Durum wheat variety Aureo (*Triticum turgidum var. durum* L.)	[58]
DNA barcoding/LAMP	Fruit juice	Pomegranate (*Punica granatum* L.), Apple (*Malus domestica* (Suckow) Borkh.), and grape (*Vitis vinifera* L.)	[59]
Whole metagenome sequencing	Different herbal products	Different taxa	[60]
Whole metagenome sequencing	Lupin seed, flour, and cookies	Lupin (*Lupinus* spp.)	[61]
Whole chloroplast genome sequencing	Dried fruit	Different species of aromatic trees (*Zanthoxylum* spp.)	[62]
Whole chloroplast genome sequencing	Berry fruit	Different berry species (*Vaccinium* spp.)	[63]
DNA metabarcoding	Honey	Different taxa	[64]
DNA metabarcoding	Honey	Different taxa	[65]

**Table 3:** List of the most recent protocols for DNA extraction from processed agri-foods and related references.

Agri-Food Matrices	Method	Reference
Must and wine	CTAB-based method/post-extraction purification	di Rienzo et al. [39]
Extra virgin olive oil	Hexane-based method	Piarulli et al. [41]
Nutmeg mace	SDS-based method	Swetha et al. [49]
Fruit juice	Filtration device	Hu and Lu [59]
Soybean oil	CTAB-based method	Xia et al. [122]
Wine	CTAB-based method	Pereira et al. [123]
Groundnut oil	DNA enrichment/CTAB-based method	Bojang et al. [124]
Honey	CTAB-based method	Soares et al. [125]
Sesame and flaxseed	SDS-based method/post-extraction purification	López-Calleja et al. [126]

## List of Abbreviations

HPLC	High-performance liquid chromatography
ELISA	Enzyme-linked immunosorbent assay
NMR	Nuclear magnetic resonance
SSR	Simple sequence repeat
SNP	Single nucleotide polymorphism
HRM	High resolution melting
RFLP	Restriction fragment length polymorphism
RPA	Recombinase polymerase amplification
LFD	Lateral flow device
LAMP	Loop-mediated isothermal amplification
GMO	Genetically modified organism
PDO	Protected designation of origin
PGI	Protected geographical indication
NGS	Next generation sequencing
WMS	Whole metagenome sequencing
